# Editorial: Aging, neurogenesis and neuroinflammation in hearing loss and protection

**DOI:** 10.3389/fnagi.2015.00138

**Published:** 2015-07-17

**Authors:** Marta Magariños, Marta Milo, Isabel Varela-Nieto

**Affiliations:** ^1^Departamento de Fisiopatología Endocrina y del Sistema Nervioso, Instituto de Investigaciones Biomédicas “Alberto Sols,” CISC-UAMMadrid, Spain; ^2^CIBERER, Unit 761, Instituto de Salud Carlos IIIMadrid, Spain; ^3^Departamento de Biología, Universidad Autónoma de MadridMadrid, Spain; ^4^Department of Biomedical Science, University of SheffieldSheffield, UK; ^5^Área de Cáncer y Genética Molecular Humana, IdiPAZ, Instituto de Investigación SanitariaMadrid, Spain

**Keywords:** ARHL, hair cells, drug delivery, lipid homeostasis, noise, redox balance, spiral neurons, TGF-β

Hearing loss affects 360 million people worldwide and it is estimated that this number will exceed 900 million by 2025 (World Health Organization. Fact sheet N°300, March 2015). Hearing loss has different etiologies; it severely affects quality of life by reducing individual communication, a fact that has different implications depending on age.

Age-related hearing loss (ARHL), also called presbycusis, is an increasing health, social, and economic problem as the affected population represents a continuously increasing percentage of the world population. Associated with ARHL is an acceleration of cognitive decline, and its links with developing neurodegenerative diseases, including Alzheimer's and frailty, are currently under study. Excessive exposure to noise and/or ototoxic drugs are additional factors in the worldwide increase of ARHL. Both noise-induced hearing loss (NIHL) and ARHL share common molecular mechanisms that involve redox imbalance and inflammation.

Sensorineural hearing loss is associated with damage or death of cochlear cells, including neurons and sensory hair cells. Hearing insults decrease cell survival pathways and promote apoptotic programs. In the first article of this eBook, “Aging, neurogenesis and neuroinflammation in hearing loss and protection,” the mechanisms behind sensorineural cell damage are reviewed (Wong and Ryan, 2015). Neuronal degeneration is typically considered secondary to hair cell loss, and another interesting article reviews the key role that innervation has on long-term hair cell maintenance (Kersigo and Fritzsch, 2015). Indeed, cochlear stressors affect not only sensorineural elements but also central components such as the auditory cortex (Fetoni et al., 2015). Similarly, aging affects the rat central auditory system in specific neuronal regions, where the expression of neurofilaments is more affected than are neuron numbers (Burianová et al., 2015). The efferent response is also altered in the senescent gerbil, indeed vestibular and cochlear efferent neurons are differentially modified (Radtke-Schuller et al., 2015). Auditory and vestibular organs have a common developmental origin (Magariños et al., 2012) and the parallel and gradual deterioration of both is strongly associated with aging. Older human adults showing hearing loss generally have increased audiometric thresholds. However, those suffering auditory deafferentation are difficult to diagnose by conventional methods. This topic explored and a novel method of diagnosis is proposed (Marmel et al., 2015).

People suffering from profound hearing loss can benefit from using hearing aids, the importance of which is being recognized by numerous awards including the 2013 Lasker-DeBakey Clinical Medical Research and the 2015 Fritz J. and Dolores H. Russ Prize Awards for the development of the modern cochlear implant. There is no other specific therapy available, but there is a boom of research efforts aimed at developing cell therapy, gene therapy, and small-molecule based pharmacological approaches. These exciting developments are based on the knowledge generated from basic neurobiology and developmental studies. Laboratory animals are essential for generating accurate models of human hearing loss. One of the articles included in this eBook describes novel models for studying NIHL, based on the use of different sound stimuli, which provide solid ground on which to study potential therapeutic molecules (Sanz et al., 2015). Another important matter that must be addressed is the delivery of potential drugs to the cochlea. The isolation and difficult access of the inner ear and the delicate balance of its internal fluids makes this problem extremely challenging. Thus, we have included an article which explores the possibilities of “smart” nanoparticles for local drug delivery (Glueckert et al., 2015).

Finally, authors herein illustrate and discuss small molecule-based novel therapies directed to confer otoprotection or reduce injury. The role of oxidative stress in hearing loss has prompted study of the potential of a combination of antioxidants and vasodilators on hearing loss remediation (Alvarado et al., 2015). Inflammation and the immune response contribute both to hearing protection as well as to hearing damage. Thus, the description of the use of TGF-β inhibitors in the protection and repair of NIHL injury (Murillo-Cuesta et al., 2015) is of particular interest. Lipid metabolism is central to neuronal degenerative diseases although its role in inner ear pathologies is essentially unknown. The role of cholesterol (Malgrange et al., 2015) and sphingosine 1-P (Romero-Guevara et al., 2015) in the physiology of hearing as well as advance future strategies based on their derivatives to fight hearing loss are explored.

## Funding

This work was supported by Spanish grant from the Ministerio de Economía y Competitividad SAF2014-53979-R and European FP7-PEOPLE-IAPP-TARGEAR. The cost of this publication has been paid in part by FEDER funds.

### Conflict of interest statement

The authors declare that the research was conducted in the absence of any commercial or financial relationships that could be construed as a potential conflict of interest.
